# The Structural and Functional Coordination of Glycolytic Enzymes in Muscle: Evidence of a Metabolon?

**DOI:** 10.3390/biology3030623

**Published:** 2014-09-22

**Authors:** Lynda Menard, David Maughan, Jim Vigoreaux

**Affiliations:** 1 Department of Biology, University of Vermont, Burlington, VT 05405, USA; E-Mail: jvigorea@uvm.edu; 2 Department of Molecular Physiology & Biophysics, University of Vermont, Burlington, VT 05405, USA; E-Mail: dmaughan@uvm.edu

**Keywords:** glycolytic metabolon, enzyme complex, muscle, metabolism, homeostasis

## Abstract

Metabolism sustains life through enzyme-catalyzed chemical reactions within the cells of all organisms. The coupling of catalytic function to the structural organization of enzymes contributes to the kinetic optimization important to tissue-specific and whole-body function. This coupling is of paramount importance in the role that muscle plays in the success of Animalia. The structure and function of glycolytic enzyme complexes in anaerobic metabolism have long been regarded as a major regulatory element necessary for muscle activity and whole-body homeostasis. While the details of this complex remain to be elucidated through *in vivo* studies, this review will touch on recent studies that suggest the existence of such a complex and its structure. A potential model for glycolytic complexes and related subcomplexes is introduced.

## 1. Introduction

Metabolism is a necessary process for the existence of life. Metabolic regulation for the purpose of increased efficiency has been imperative for the evolution of living organisms and strategies have gone beyond allosteric regulation and enzyme or substrate concentration to covalent modification, compartmentalization, and the specialization of organelles [1,2,3]. Physical and chemical coordination is inherently linked to the metabolic capability and the well-being of the organism, such that the orchestration of metabolic processes has been a driving force in evolution and organismal success.

Increased biological complexity has been associated with both increased temporal and spatial organization of biochemical processes. The most primitive organisms have had to accommodate metabolic functions that would be redundant if not appropriately controlled, minimize detrimental side-reactions, or sequester energy-rich products while separating them from toxic side-products. It is because of this that separation of function by compartmentation has been a characteristic accompaniment to all life, possibly as far back as the primordial cell [4]. Nature has demonstrated favoritism for the compartmentation of cellular processes through the development of organelles and the evolution of originally multipurpose compartments, such as chloroplasts and mitochondria, to more specialized operations. Localized assignments have been evident within membrane-restricted structures via binding to trans-membrane proteins [5], structural networks of the cytoskeleton [6], and steric aversion or attraction to other proteins or along ionic gradients [7].

**Figure 1:**
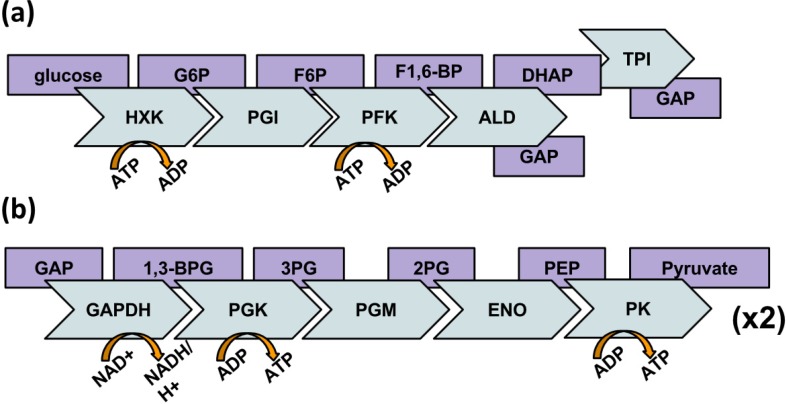
Glycolysis, in which blue chevrons represent the glycolytic enzymes and purple rectangles represent substrates. (**a**) shows the hexose portion of the glycolytic pathway in which 2 ATP are consumed per glucose. (**b**) shows the triose portion of the glycolytic pathway that proceeds after glucose is split in to two Glyceraldehyde-3-phosphates (GAP) whereupon 4 ATP and 2 NADH are produced. Abbreviations: Glucose-6-Phosphate (G6P); Fructose-6-Phosphate (F6P); Fructose-1,6-Bisphosphate (F1,6-BP); Dihydroxyacetone phosphate (DHAP); Glyeraldehyde 3-Phosphate (GAP); 1,3-Bisphosphoglycerate (1,3-BPG); 3-Phosphoglycerate (3PG); 2-Phosphoglycerate (2PG); Phosphoenolpyruvate (PEP); Hexokinase (HXK); Phosphoglucose isomerase (PGI); Phosphofructokinase (PFK); Aldolase (ALD); Tripsephosphate isomerase (TPI); Glyceraldehyde phosphate dehydrogenase (GAPDH); Phosphoglycerate Kinase (PGK); Enolase (ENO); Pyruvate Kinase (PK).

Glycolysis (Figure 1) is an enduring process of anaerobic metabolism for all organisms, save for lithotropes, and the involved enzymes have been suspected for many years to engage in kinetically-relevant complex formation. The Protozoan *Trypanosoma brucei* has a glycosome, specific membrane-bound organelle dedicated to glycolytic enzymes, as evidenced initially by fractionation [8]. The existence of this organelle is an indication that physical compartmentation of glycolysis is advantageous [9]. In this organism, rates of glycolysis are 50 times that of mammalian cells as *T. brucei* relies almost entirely, perhaps solely, on glycolysis when in its mammalian host [10]. This necessitates the more intense maintenance of the pathway. A level-down in compartmentation, as might be observed in mammalian cells requiring glycolysis even if not solely, may be dynamic complex-formation among these enzymes. The hypothesis that glycolytic enzymes form localized multi-enzyme complexes is not a new one [11,12]. This putative glycolytic complex, although in line with the cell’s propensity for organization and consistent with considerable circumstantial evidence, has evaded direct experimental support [13,14] and is subject to controversial interpretations [15]. Here, we review studies over the past quarter century that examine the existence of a glycolytic enzyme complex. We also present a possible model of this “glycolytic metabolon” based on available experimental evidence attained from literature published in 1990 onwards.

## 2. Behavior of Glycolytic Enzymes

Dynamic glycolytic enzyme complexes allow intricate regulatory control. Seven notable modes of regulation for glycolysis that have been reported include (1) classic substrate saturation; (2) cofactors and ions; (3) competitive or non/uncompetitive inhibitors; (4) positive and negative allosteric effectors; (5) dissociation, association and self-association; (6) chemical interconversion and (7) changes in enzyme concentration and ratios by synthesis and degradation [13]. In *Saccharomyces cerevisiae*, a larger number of subunits of glycolytic enzymes are associated with more intricate control reflected in the level of binding cooperativity represented by the Hill coefficient [13]. That is, an increase in the number of subunits allows for greater regulatory control. By extension, one can envision a complex of multiple sets of subunits evoking further means of regulation and combined functionality.

Dynamic association of glycolytic enzymes can regulate the metabolic pathway, partially or as a whole. As complexes shift in orientation or binding partners, the quantity and type of regulatory sites available may also shift. The need for enhanced regulation of the glycolytic pathway is the major reason why *T. brucei* developed a glycosome. During certain life-cycle stages the organelle itself can be discarded [9,16]. *T. brucei* is unique in possessing a glycolysis-specific organelle, but the existence of such an organelle encourages the notion that different strategies of compartmentation of glycolysis may occur in other organisms. It must also be considered that localization and regulation of individual enzymes may vary not only between organisms but also between cell types [13].

Glycolytic enzymes, long identified as soluble proteins of the cytoplasm, have been found to be ambiquitous (defined as having the ability to be distributed either on a structure or dispersed within a solution) depending on cytosolic conditions [17]. Many of the glycolytic enzymes have been proposed to bind cellular structures, as evidenced by their presence in insoluble cellular fractions [18,19,20,21,22]. For example, studies in cardiac and skeletal muscle have shown localization of glycolytic enzymes to the ATPase calcium pumps on the sarcoplasmic reticulum [23,24]. Glycolytic enzyme complexes are likely to be transient depending on the energy needs and the metabolic state of the cell, dictated by concentrations of the cycling of effectors, substrates, pH and other variables. Inconsistency in detection of glycolytic enzyme assemblies stems from the incorporation of variable cell conditions among experiments and difficulty mimicking physiological conditions *in vitro* that may influence dynamic compartmentation of the glycolytic enzymes [2].

*In vivo* evidence of pools of cellular ambiquitous glycolytic enzymes has been supported by studies with permeabilized cells. For example, dextran sulphate-permeabilized mouse fibroblasts were stimulated to engage in glycolysis and it was found that glycolysis continued with no glycolysis taking place in the extracellular environment [25]. In this case, the active glycolytic enzymes were retained within the permeabilized cell, suggesting that not all glycolytic enzymes were in a freely diffusible state. Further support of partial retention was evidenced in saponin-permeabilized CHO cells that were used to investigate diffusion of molecules from cells still possessing intact structural components. These cells were compared to saponin-permeabilized cells structurally compromised by treatment with latrunculin B, which specifically sequesters actin monomers, thus stopping actin polymerization [26]. It was found that a maximum of 25% of the total protein was released in the saponin-permeabilized cells compared to 70% of the total protein in latrunculin B-treated saponin-permeabilized cells. Glyceraldehyde 3-phosphate dehydrogenase (GAPDH) and Lactate dehydrogenase (LDH) were readily released from saponin-permeabilized cells with an additional release from latrunculin B-treated cells. More recently, a study employing a novel microvolumetric technique to evaluate diffusible proteins of muscle was utilized to examine the stoichiometry and diffusion properties of glycolytic enzymes [27]. A 1:2 stoichiometry of glycolytic enzymes of the hexose pathway: triose pathway was found, reflecting the flux differences in substrate intermediates, a finding consistent with the existence of a glycolytic complex.

### 2.1. Moonlighting Functions

The original assumption of a diffuse well-mixed cytoplasm has been dismissed and it is now widely accepted that the cellular milieu is organized out of necessity [28]. A major advantage to a compartmentalized cytoplasm becomes evident when considering ‘moonlighting’ functions of many intermediates and metabolic enzymes. Glycolytic enzymes have been identified to be involved in a variety of non-glycolytic functions, even extracellularly [29]. GAPDH is a well-researched example [30]. GAPDH has been implicated in regulation of gene expression [31], apoptosis [32,33], and even playing a role as a cell surface receptor [34]. Having one protein responsible for a variety of functions is conservative and efficient on the part of the organism, but requires locational organization to keep these functions in check.

It has been suggested that glycolytic enzymes have stabilizing effects on cytoskeletal elements such as actin and tubulin, and may even turn the cytoskeleton into a metabolic sensor [35]. The notion of a metabolic sensor follows directly from considering how an availability of nutrients is able to regulate the cell cycle and cytokinesis. Metabolites and intermediates may also possess alterative functions or be involved in regulating moonlighting functions of the corresponding enzymes. In such cases, transient and dynamic complexes could accommodate proteins that are engaged in extremely important, well-regulated, but variable, functions.

In the following sections, we propose a hypothetical model for a dynamic and active glycolytic complex through the initial formation of subcomplexes. In proposing such a model it is important to keep in mind that complexes may serve more than one function and that dysregulation at this level may be behind many pathologies [36,37]. The fundamental importance of understanding enzymatic complexes, especially those involving metabolism, is widely recognized. Metabolism has been considered to be a founding factor in the prebiotic world where microcompartmentation in protocells may have further driven the evolution of life [38]. Microcompartmentation, either due to membrane-restriction or by clusters of enzymes interacting with each other to localize their activity—such as in a complex—may even have further implications or incorporate functions that are currently unrecognized or not fully understood. Understanding the structure of glycolytic subcomplexes and their assemblies, through hypothesis and experiment, is an important starting point for unravelling the multi-functional sub-structure of cells relying of anaerobic metabolism for their energy needs—such as fast twitch cells with their highly evolved, highly organized internal structures.

### 2.2. Implications of a Glycolytic Enzyme Complex

In addition to greater regulatory control, a glycolytic complex would promote at least two other beneficial conditions for the cell: (i) increased solvation capacity with the cytosol and (ii) channeling of substrates. Compartmenting metabolic pathways via complexes may be imperative since the concentration of protein in the cytosol is close to the concentration at which detrimental protein crystals form [39,40]. The channeling promoted in a complex would permit a kinetic advantage [41] in addition to preserving the cytosol solvation capacity by limiting substrate diffusion.

Creating enzyme complexes to permit a greater solvation capacity is advantageous for regulating macromolecular crowding, which modulates protein folding, aggregation and mobility [42]. Just as pathological alternations in protein density can be detrimental, alterations in protein crowding, if tightly regulated, can be advantageous. This can be achieved by promoting beneficial metabolic pathways that are not constitutively present. An appropriately timed increase in crowding, such as that which occurs in proliferating cells, will actually promote the binding of glycolytic enzymes to cytoskeletal structures [42]. This has been recognized especially for phosphofructokinase (PFK), enolase (ENO), and pyruvate kinase (PK) [43]. Crowding has been shown to greatly influence the conformation of phosphoglycerate kinase (PGK), where Fluorescence Resonance Energy Transfer (FRET) was used to identify two alternatively stabilized PGK conformations that had a major positive effect on enzymatic activity [44]. Thus, the formation of specific complexes may serve a role in regulating macromolecular crowding effects in addition to serving a metabolic function.

The kinetic advantage of channeling is in the decreased transfer time of metabolites between enzymes without having to equilibrate in the bulk solution of the cytosol, allowing for accelerated establishment of steady states [45]. It has been estimated that 80% of the metabolic intermediates of a cell serve only one purpose such that diffusion into the cytosol may be considered wasteful [14]. It has also been suggested that there is not enough water in the cell to permit optimal concentrations of all intermediates [46] assuming that complex formation and channeling does not occur. Enzymes or, specifically, active site concentrations have been suggested to exceed substrate concentrations in the case of glycolytic enzymes, primarily for muscle enzymes [47,48], which would be characteristic of complex formation involving the channeling of substrates.

### 2.3. Muscle: An Ordered Model for Metabolism

Most members of the kingdom Animalia, the most species-abundant of eukaryotes, heavily rely on muscle for survival. In mammals, muscle represents the body’s largest reserve of protein and stores the bulk of the body’s glycogen. It is highly involved in regulation of electrolytes, pH and glucose homeostasis. In humans, skeletal muscle is the largest organ, making up 40%–50% of total body mass [49,50]. It is the largest insulin-sensitive tissue in the body and does not export glucose, utilizing all of its glucose and glycogen for its own function. Muscle is extremely adaptive to changes in energetic demand and dynamic metabolic flexibility is essential to its functional efficiency. In this way, the functional purpose and adaptation of muscle is defined by its metabolic profile.

Skeletal muscle has been recognized as integral to metabolic health and has been implicated in numerous issues relating to both whole body and localized metabolic health [51]. Metabolic inflexibility is characterized by an inability for skeletal muscle to transition between energy sources and is specifically linked to impaired glucose uptake and usage [52]. Upon insulin stimulation, skeletal muscle utilizes 75% of the body’s glucose, making it a clear focus of study when considering metabolic diseases such as diabetes [53]. Metabolic syndrome, affiliated with type 2 diabetes and obesity, has been associated with a decreased volume of oxidative muscle fibers, reduced glucose transport, and down regulation of enzymes involved in oxidative metabolism, but with increased presence and activity of glycolytic enzymes in the skeletal muscle of obese subjects [54,55]. Significantly increased expression of GAPDH and ALD, along with adenylate kinase, a known regulator of glycolytic enzymes, was identified in obese individuals over lean controls [55]. An increased reliance on glycolysis over oxidative metabolism is identified in these cases of obesity both with and without diabetes. Other metabolic diseases, such as in the aerobic metabolic disorder Myalgic Encephalomyelitis, also known as Chronic Disease Syndrome [56], may require patients to rely on glycolysis, perhaps more than that required of healthy individuals, to compensate for the mitochondrial dysfunction that underlies the disease [57]. It is possible that up-regulation of glycolysis via regulatory mechanisms embedded in the enzyme complex may play a role in ameliorating the disease symptoms [58].

Skeletal muscle consists of extremely well-ordered myofibrils, composed of hexagonal arrays of closely packed, interdigitating thick and thin filaments, bundled in repeating sarcomeres, from one end of the muscle to the other. The sarcomere is a dynamic network of proteins with metabolic enzymes playing a major role in cellular homeostasis, contractile function, and remodeling [59]. The sarcomere represents a compact, ordered functional unit that must accommodate variable conditions and control metabolism in a dense environment. Its primary purpose of force generation and muscle shortening, consistent among Animalia, requires reliable and flexible tuning of critical metabolic processes. Thus, muscle is an excellent model in which to understand the interaction of metabolic enzymes coordinating with well-defined structural components, particularly in relation to normal function and metabolic diseases.

In general, cellular metabolic processes appear to be reaction controlled and not limited by diffusion [54]. Coordination of substrate availability and spatial constraints occurs in spite of the high volume occupancy of cellular proteins [60]. In muscle, the cellular proteins consist primarily of structural cytomatrix proteins; mainly myosin and actin, the primary constituents of the thick and thin filaments, and cytosolic proteins; in fast twitch skeletal muscle, mainly glycolytic enzymes, phosphocreatine kinase, and parvalbumin [27]. Even within the crowded environment of a muscle cell, however, the production and transport of metabolic substrates and products appear to be highly efficient, highly regulated, and optimized through evolutionary processes.

Although many studies report indirect evidence of glycolytic enzyme complexes, few studies have sought evidence *in vivo* or under conditions that closely mimic those *in vivo*. One study that approximates the latter was published recently [58]. In that study, the efflux time course of glycolytic enzymes diffusing from demembranated muscle fibers into a physiological salt solution were consistent with the existence of complexes at the onset of diffusion, which subsequently dispersed. Other studies have been limited by examination of only two or three enzymes, or have experienced challenges comparing data and corresponding structural models derived from different experimental conditions and sources (including yeast, bacteria, plants, and various mammalian cancer cell lines). While the studies as a whole provide a basis for speculation, additional studies are needed to demonstrate unequivocally the existence of glycolytic enzyme complexes *in vivo.* Muscle, with its centrality to organism metabolism and consistent organization among organisms, stands out as a useful model to pursue such further study in pursuit of the elusive glycolytic enzyme complex.

## 3. A New Hypothesis on Glycolytic Enzyme Complexes

Kurganov *et al.* (1985) [10] reviewed the experimental evidence for binding between glycolytic enzymes and were the first to propose a nearly all-inclusive glycolytic complex. The formation of a glycolytic complex, excluding only hexokinase (HXK), was proposed to take place in three stages. According to Kurganov *et al.*, the glycolytic enzymes do not first form subcomplexes but join the main complex in waves. It was postulated that once PFK is bound to a support (e.g., erythrocyte membrane or F-actin), PK and GPI would bind; followed by aldolase (ALD), GAPDH, LDH; and finally, by PGK, PGM, ENO, TPI and glycerol-3-phosphate dehydrogenase (GPDH). However, support for “Kurganov’s complex” gradually eroded in light of further studies [61].

More than a quarter-century later, with the improvement of techniques and accumulation of better controlled experiments, we may be in a better position to deduce, and model, a glycolytic metabolon. In particular, the possibility of subcomplexes within this –“glycolytic complex” has been raised, in which separate groups of glycolytic enzymes from different sections of the pathway interact [62]. These subcomplexes may then either interact, directly or indirectly, with each other. The existence of subcomplexes prior to forming a whole complex may represent regulated subsections that may have greater stability, or a more defined presence, compared to the full complex.

Table 1 shows the glycolytic enzymes from PFK to PK, indicating their reported relationships with one another, noting by symbols whether there is recent (>1990) *in* *vitro*, *in vivo*, or *in silico* data suggesting direct interactions between them or with F-actin. Reported propensities for self-aggregation have been omitted. Taking into consideration whether or not these interactions involve activation or deactivation of enzymatic activity, or stabilization, we hypothesize that the complete, active enzyme complex consists of three subcomplexes. The summary table leaves out HXK and PGI due to a lack of data supporting interaction with other glycolytic enzymes. The table also excludes results from yeast. Both *in vitro* and *in silico* studies imply that not all glycolytic enzymes of *S. cerevisiae* (a widely used model) possess binding profiles comparable to those from mammalian systems. This caveat has been important in interpreting and comparing what otherwise may be considered confounding, if not contradictory, results from mammalian and yeast studies.

There is a distinct lack of evidence for HXK binding within the sarcomere, some evidence of HXK binding to membranes [63,64,65] and *S. cerevisiae* HXK binding other *S. cerevisiae* glycolytic enzymes and actin [66]. HXK also binds to mitochondria [67,68]. The few studies examining its interaction with actin, conducted in plants, curtail their findings, noting that it is primarily a mitochondria-associating enzyme [69,70].

**Table 1:** Interactions of glycolytic enzymes from post-1990s literature, excluding *S. cerevisiae*. A green circle is indicative of modelling evidence, a blue square is indicative of *in vitro* studies and an orange diamond is indicative of *in vivo* bacterial studies [68]. Color intensity is indicative of the evidence supporting an interaction: light intensity, one to two published studies supporting an interaction; dark intensity, three or more published studies supporting an interaction. Self-interactions are not shown.

Ligands	PFK	ALD	TPI	GAPDH	PGK	PGM	ENO	PK	F-actin
**PFK**		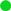 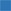	▪	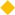	▪	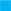 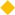	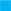 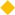	▪	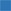
**ALD**	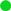 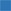	▪	▪	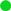 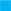	▪	▪	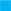	▪	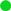 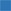
**TPI**		▪	▪	▪	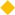	▪	▪	▪	▪
**GAPTH**	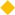	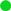 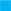	▪	▪	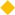	▪	▪	▪	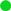 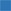
**PGK**	▪	▪	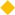	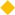	▪	▪	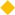	▪	▪
**PGM**	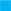 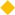	▪	▪	▪	▪	▪	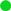 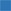 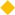	▪	▪
**ENO**	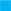 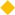	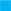	▪		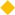	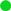 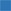 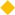	▪	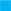 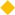	▪
**PK**		▪	▪	▪	▪	▪	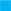 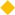	▪	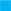

The case against PGI is not as strong as that against HXK, but is still substantial. Unlike HXK, there had been some early studies that were suggestive of PGI binding to muscle F-actin [11]. One more recent study found that PGI immunoprecipitated with actin, but only in *S. cerevisiae* [66]. Studies including most of the glycolytic enzymes have left out PGI [71], have used it as a control ‘soluble’ enzyme [72] or simply did not identify PGI binding F-actin or other glycolytic enzymes even with more generalized searches for binding partners [73,74].

### 3.1. Glycolytic Subcomplex-1 (GlySCx1): PFK, ALD, & GAPDH

The first of the three subcomplexes incorporates two well-studied glycolytic enzymes in complex formation: ALD and GAPDH. Both enzymes have been localized to the sarcomere in muscle [71,75]. There have been multiple *in vitro* studies supporting GAPDH binding to actin [76,77,78] and ALD binding to actin and/or GAPDH [72,76,79,80,81,82]. The general consensus is that ALD binds F-actin predominantly in an inactive state and close to its active site. Upon substrate binding, its conformation becomes unfavorable for actin binding and it dissociates [83,84]. Similar to ALD, actin-bound GAPDH [85] and tubulin-bound GAPDH [86] has been suggested to be inactive. There is no indication whether or not activity levels of GAPDH are affected when GAPDH is bound to ALD.

Several studies have relied on Brownian dynamics (BD) simulations to model possible interactions between glycolytic enzymes, more specifically between GAPDH and ALD, and with actin [25,78,87,88,89,90,91]. This method models factors in concentration of solutes and electrostatic forces, while modeling the relative rotation and diffusion of whole molecules. The electrostatic potential around each protein was calculated alongside x-ray structures for the development of a charge-mapped model for each of the proteins examined. Modeling of ALD binding to actin was enacted to clarify what residues may be involved in an electrostatically-based association [85]. ALD residues previously suggested to bind actin include residues A32 through E52 [92], and catalytic site residues R42, K107 and R148 [83]. Actin residues that bind ALD include D24-D25 and E99-E100 [93]. These studies were followed by further evaluation of ALD binding to GAPDH with the consideration of substrate channeling [89] and examination of the propensity for GAPDH to bind actin [90].

*In situ* investigations concerning ALD and actin led to the identification of two large regions of positive potential that present themselves through ALD’s quaternary structure that could bind subdomain 1 of either G-actin or F-actin [91]. It was determined that seven ALD tetramers could bind 13 actin subunits on F-actin. ALD A32-E52 was not found to be a region of substantial electrostatic potential and the catalytic residues R42, K107, and R148 were not accessible according to *in situ* studies [91]. Actin residues D24-D25 and E99-E100 were found to have binding potential. Although ALD is suspected to be inactive when bound to F-actin, there is no evidence suggesting activity is affected when bound to GAPDH. This brings forth the possibility of multiple states for glycolytic complexes such that binding may be taking place in either an active or inactive form, but in different ways. In this paper, we will only be considering a potentially ‘active’ complex that is supported by the studies summarized in Table 1.

In a subsequent article, an interaction between GAPDH and ALD was evaluated and glyceraldehyde 3-phosphate (GAP) was modeled as moving out of the active site of ALD [89]. While GAPDH and ALD are engaged in favorable intermolecular contacts, the efficiency of GAP transfer was compared to that of GAP migrating from solution. The most common interaction between ALD and GAPDH was observed to be between two adjacent subunits of GAPDH and two subunits of ALD. An advantage in efficiency of GAP transfer was observed in the channeling experiment.

Binding of GAPDH with actin was examined by BD modeling [90] and was found to have numerous smaller patches of electrostatic potential compared to ALD, which possessed larger regions of positive potential and, as a result, engaged in more stable interactions with actin. As with ALD, actin subdomain 1 was predominantly engaged in contacts with GAPDH with the most stable complex occurring with one actin monomer binding one subunit of GAPDH. Interaction between two actin subunits and three GAPDH subunits was also observed.

BD modeling has been used to compare *S. cerevisiae* and muscle ALD and GAPDH binding to *S. cerevisiae* and muscle actin [78]. It was found that, while muscle actin and muscle GAPDH and ALD show affinity for each other, yeast actin had a greatly decreased affinity for both muscle and yeast ALD and GAPDH. When paired with muscle actin, yeast ALD would not bind and yeast GAPDH had a significantly lower affinity than muscle GAPDH. Muscle ALD from zebra fish and human were then examined for actin binding to make comparisons among species [88]. Binding sites were found to be conserved among species but notably missing in the case of *S. cerevisiae* proteins. This may provide some insight into why a number of *in vitro* studies involving *S. cerevisiae* F-actin binding to glycolytic enzymes has been less successful than with the vertebrate counterparts. Vertebrate ALD and GAPDH interactions with actin have been corroborated, through recent BD calculations, simulated at various ionic strengths, including physiological [87].

The third enzyme of this first subcomplex is PFK, one of the most highly regulated enzymes of the glycolytic pathway. Studies have suggested that PFK can bind GAPDH [74] and ALD [1,94,95], although the evidence for this binding is not as strong as the evidence supporting the ALD-GAPDH interaction. Interestingly, PFK dimers have been found to be able to be bound and activated by ALD when PFK dimers would otherwise be inactive [94]. This introduces the interesting possibility that, when involved in a complex, PFK may be in a dimer form, rather than a tetramer. PFK has been found to have full activity when bound to F-actin as well [96,97] with the bound fraction increasing upon activation of glycolysis [98,99,100].

**Figure 2:**
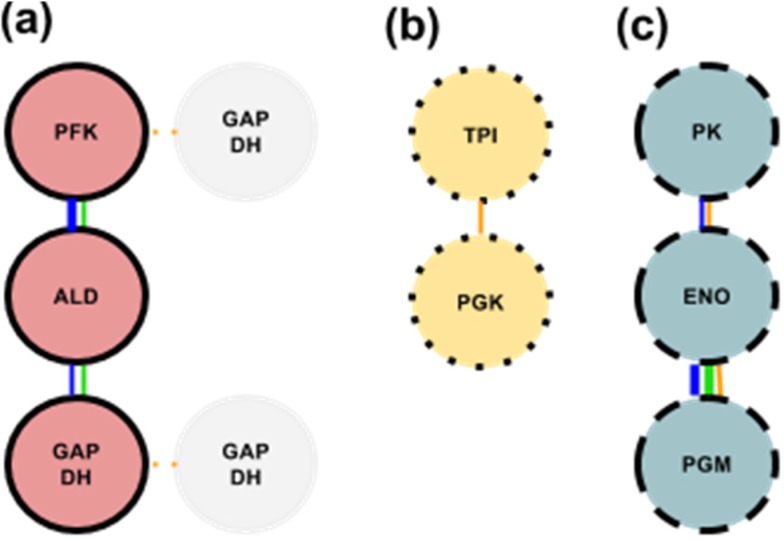
(**a**) GlySCx1 denoted by intact black borders and red interior with the second possible GAPDH in gray at the two possible positions, (**b**) GlySCx2 with members denoted by dotted borders and yellow interior, (**c**) GlySCx3 with members denoted with dashed borders and blue interior. Connecting lines represent a specific interaction between the enzymes with the color relating to the type of study that has shown an interaction (see Table 1 for color code). Interactions that are supported by three or more studies are indicated with a thick line and interactions that are supported by less than three studies are indicated with a thin line. Each circle represents an entire functional enzyme (subunits included).

Based largely on these studies, we propose glycolytic subcomplex-1 (GlySCx1) (Figure 2a). The active form of this complex has PFK bound to both F-actin and ALD. ALD is additionally bound to GAPDH. These interactions have the most support even though other interactions have been reported (Table 1). The inactive forms of these enzymes could be localized nearby due to the repetitive nature of F-actin. Because the actin-binding site for these three enzymes is the same, with active ALD having a decreased affinity for F-actin and active PFK having an increased affinity for F-actin, it would follow that active PFK could easily displace active ALD when competing for the same binding site. This is further encouraged by the idea that PFK could bind inactive F-actin-bound ALD first and then displace it from F-actin with both of them in their active conformations.

### 3.2. Glycolytic Subcomplex-2 (GlySCx2): TPI & PGK

The second glycolytic subcomplex (GlySCx2) is proposed to be composed of TPI and PGK (Figure 2b). Evidence for the binding between TPI and PGK comes from cross-linking studies in bacteria [74]. Another study on the hyperthermophilic bacteria *Thermotoga maritime* demonstrated a covalently-linked complex between TPI and PGK, an interaction proposed to improve enzyme stability [101]. Another study showed sarcomeric co-localization of TPI and PGK in Drosophila flight muscles [71] at the M band and Z disc. However, TPI is known not to bind F-actin from *in silico* modeling [87,102] and *in vitro* studies [78] and there is no evidence whether PGK does or does not bind actin.

The only support of TPI binding any other glycolytic enzyme is for PGK (Table 1). In the case of PGK, however, there appears equally weighted support for PGK binding TPI, GAPDH, and ENO. This necessitates examination of GAPDH and ENO’s separate support for alternative binding partners. Unlike the case for a TPI-PGK interaction, both GAPDH and ENO possess more support for binding other glycolytic enzymes over PGK. Since their interaction with non-PGK-associated enzymes is better supported, we did not consider GAPDH and ENO to be members of GlySCx2.

GAPDH and ENO have equivalent support for a PGK interaction when considering the PGK support alone, but this changes when taking into account the rest of GAPDH and ENO’s support profiles. Of the five enzymes that ENO has been reported to have affinity for, support for an ENO-PGK interaction is the weakest and, because of this, binding between PGK and ENO is dismissed due to a relative lack of likelihood. GAPDH’s support profile is composed of fewer potential binding partners and fewer studies backing those interactions. In the case of GAPDH, support for a PGK-GAPDH interaction is not completely dwarfed by support for other glycolytic enzyme interactions. It is because of this that an interaction with GAPDH is considered later when considering formation of a full complex.

### 3.3. Glycolytic Subcomplex-3 (GlySCx3): PGM, ENO, & PK

The third proposed subcomplex is composed of PGM, ENO and PK (Figure 2c) and possesses some unique characteristics that differentiate this complex from the other two, with a greater emphasis being on tubulin or troponin binding over actin binding. There is little recent data suggestive of F-actin binding to any of GlySCx3 enzymes. There have been *in vitro* studies suggesting that ENO and PGM specifically do not bind actin [78,103] and limited recent studies of PK involving the binding of F-actin outside of *S. cerevisiae* [80]. Both PK and PGM have been found to bind ENO [97]. These three enzymes appear to bind to a common site in tubulin and in troponin [104,105,106,107,108]. PGM has been found to be localized to the sarcomere in Drosophila flight muscle [65] and vertebrate skeletal muscle [71,75]. ENO was also found localized to vertebrate skeletal muscle sarcomeres at the M line [108,109].

In the GlySCx3, ENO serves as the enzyme that links PGM and PK. Studies *in silico* suggest binding near the active sites and channeling between mammalian PGM and ENO (also demonstrated for *S. cerevisiae* enzymes) [110,111,112]. These findings have pointed to the C-terminal region of PGM as the location of binding to ENO, specifically Ala239 and Val240.

PGM and ENO have been hypothesized to share troponin binding affinities with PK, laying claim to a potential binding site on thin filaments that might be very similar to that of GAPDH [74]. Other possible interactions that have been suggested for members of this complex is PGM interacting with PFK [74] or GAPDH [113] and ENO interacting with PGK, PFK [74] or ALD [103]. The many possible interactions for the enzymes of GlySCx3 lead us to propose that GlySCx-3 may proceed with one of three outcomes: (i) GlySCx3 localizes to GlySCx1-2 by one or more of its three enzymes binding troponin adjacent to the F-actin bound GlySCx1, (ii) GlySCx3 localizes to GlySCx1-2 by a link to F-actin by PK or (iii) GlySCx3 binds an enzyme of either GlySCx1 or GlySCx2.

### 3.4. Formation of the Glycolytic Complex

These three putative subcomplexes appear feasible based on the studies that have been published; nevertheless, the subcomplexes may be interacting in ratios other than 1:1:1. In fact, this seems extremely likely. Glycolysis in muscle has been shown to incorporate twice the molar ratio of enzymes in the triose phase as the hexose phase [27]. This doubling of enzymes for the triose part of the pathway supports channeling [114]. This is expected given the shift from the hexose portion of the pathway to the triose portion of the pathway whereupon intermediates double. PFK first binds F-actin, possibly displacing an inactive form of ALD. After this, ALD binds PFK and GAPDH binds ALD. GAPDH is on the outskirts of this trifecta and there is the possibility of there being two GAPDH involved, either by its own natural capacity for self-association or by binding PFK [74].

Here, GlySCx1 is proposed to be responsible for binding and localization of GlySCx2 to the thin filament through GAPDH-PGK interaction. Interaction between PGK and GAPDH is supported by crosslinking studies [74]. The idea is further supported by previous data suggesting that TPI localization in muscle is dependent on ALD and GAPDH [115] that was later re-confirmed along with PGK [62]. Depending on the orientation of PGK binding GAPDH, the orientation could permit TPI, though not binding GAPDH directly, to have a localized benefit for the transfer of GAP to GAPDH. In such a case, it would follow that two GlySCx2 could bind two separate GAPDHs.

**Figure 3:**
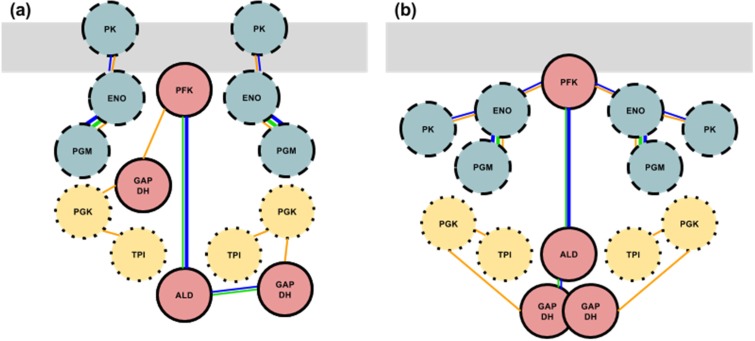
Two possibilities for how the three subcomplexes could form a full, active glycolytic complex. The members of the triose pathway are doubled in accordance with previous stoichiometric experimental studies [23]. Each circle represents an entire enzyme (*i.e.*, all subunits). The grey bar represents a thin filament composed of F-actin, troponin and tropomyosin. The three subcomplexes are designated by their appropriate borders and colors (see Figure 2). Lines connecting the enzymes indicate a specific interaction as described in Figure 2. Physical contact of GAPDH represents self-binding.

Figure 3 shows a two-dimensional interaction map of two proposed formats for a glycolytic enzyme complex consisting of the three proposed subcomplexes. In both possibilities, all three subcomplexes are in their proposed binding arrangements amongst themselves and PFK is anchored to F-actin. There is one GlySCx1 per every two GlySCx2s and two GlySCx3s in the final complex. In the first format (Figure 3a), PFK is additionally binding GAPDH and GlySCx3 is not directly bound to either GlySCx1 or 2 but is bound to either actin or troponin components of the thin filament by PK. In the second format (Figure 3b), ENO is linking GlySCx3 to PFK and both GAPDH enzymes are still linking two GlySCx2s to the GlySCx1. This second format is an example of how GlySCx3 may be bound to GlySCx1 by ENO, but this connection, for instance, could also be formed with PGM. The positions of these enzymes promote channeling and an important note is the vicinity of PGK with PGM and TPI near ALD and GAPDH. TPI and PGK are not sequential in the glycolytic sequence but are shown binding each other here such that both of them are brought into close enough proximity for channeling to be possible.

## 4. Conclusions

The research to date has not been sufficiently consistent to establish, unequivocally, the structure of a glycolytic complex. Part of the problem is that the experiments have been subject to a wide variety of conditions. The lack of solid evidence highlights the need for progress in this area. Most published reports involving the interaction of the glycolytic enzymes with each other or bound to F-actin or troponin supports the idea that direct contact takes place among them, even if the reported evidence is not concrete. Not all of the glycolytic enzymes, however, have been researched to the same extent or have been reconfirmed in the same model organism. In muscle, many enzyme–enzyme interactions have not been examined or have been looked at only indirectly; the largest weight so far can only be put on suggestive subcellular co-localization and stoichiometric studies. Physical associations among glycolytic enzymes are generally not supported by protein interaction databases that include high-throughput experiments, such as MINT, and STRING. There is a dire need to examine the complex in an *in vivo* context to fully develop an accurate picture. Such an understanding is critical to the way we think about metabolic activity, regulation and its role in pathologies.

Admittedly, there has been few *in vivo* studies promoting a glycolytic enzyme complex have been published to date. However, as new methodologies and model systems are developed, breakthroughs in understanding the complex structure and function of the glycolytic enzyme system are more likely. Studies incorporating muscle preparations will continue to be important contributors but inspiration can be gleaned from *in vivo* work done in other cell types. In cancer cells, knockdown of ALD was shown to inhibit proliferation by 90% and yet the phenotype could be rescued by an enzymatically nonfunctional ALD still able to bind F-actin [116]. The implication that binding and catalytic attributes of glycolytic enzymes are not obligatorily linked presents several possibilities. The catalytic sites and sites of binding are mutually exclusive on the level of secondary structure and thus can be examined separately. Additionally, there may be distinct functional, but not enzymatically active, enzyme organizations that could be maintaining an advantageous localization for alternative functions.

*D. melanogaster* represents a suitable model for examination of muscle glycolytic enzyme complexes. Sarcomeric co-localization of glycolytic enzymes in *D. melanogaster* IFM support continued used of this model organism to elucidate the nature of a glycolytic enzyme complex [71,117]. Anaerobic metabolism plays a role in dipteran flight even though the indirect flight muscle (IFM) of *D. melanogaster* is highly oxidative. The *D. melanogaster* IFM is a well-understood muscle model that experiences very high energetic demands and has been implicated in having well optimized glycolytic flux such that pH within the muscle remains the same throughout the continuous rapid contractions associated with flight [118]. The remarkable efficiency of glycolysis in the IFM is highlighted in a study which used full and partial P-element excision-derived mutagenesis to examine flies experiencing specific glycolytic enzyme deficiencies [119]. The study found that even very low expression of certain glycolytic enzymes were unrestrictive to wing beat frequencies suggesting that factors other than enzyme concentration, e.g., formation of a complex for effective substrate canalization, contribute to maintain metabolic capacity.

Glycolytic enzymes are considered required for viability for many organisms such that developing viable nulls or other mutations may be feasible only in model genetic organisms, such as *Drosophila*, that allow regulated, inducible expression [120]. Selecting alternative isozymes as was done for GPDH [113] is another investigative approach, along with replacing a glycolytic enzyme of one organism with a functional alternative from a different species. For example, PGI and ALD of *S. cerevisiae* was replaced by the corresponding enzymes in *E. coli* and *D. melanogaster*, and glycolytic functionality was not found to be compromised [121]. The propensity for *S. cerevisiae* enzymes binding structural elements is substantially less than for that of their muscle counterparts [66,78,102]. This implies that transgenically exchanging muscle enzymes in *D. melanogaster* for the corresponding yeast enzymes may serve as an *in vivo* means for elucidating glycolytic enzyme or F-actin interactions. Experiments using zero-length UV-reactive crosslinking may be another way to examine complex formation in *D. melanogaster in vivo*. For example, this method could be used to induce crosslinking during flight to capture complexes that may form in such transient and fluctuating conditions.

The proposed model of three subcomplexes (Figure 2) is reasonably supported by research accumulated from studies involving bacterial and mammalian cell models, molecular dynamics modelling, kinetic and stoichiometric experiments, and sub-cellular localization. Examining how these possible individual subcomplexes interact or, how the members of each subcomplex interact with each other *in vivo*, may be a prudent first step. One can examine how directly a certain subcomplex is engaged with the other subcomplexes by ablating that specific subcomplex and observing the effects, quantitatively and qualitatively, on the other enzymes of the other two subcomplexes. For instance, if GlySCx3 is simply localized to GlySCx1 and 2 by binding to troponin (Figure 3a), then ablation of GlySCx3 will have little to no effect on the function and localization of GlySCx1-2. In such a case, ablation of GlySCx1-2 would also have no effect on the binding and localization of GlySCx3 to thin filament troponin. However, in an alternate scenario where GlySCx3 is localized to the active complex by PFK and ENO binding (Figure 3b), then disruption of the binding site on either PFK or ENO should disrupt the entire GlySCx3.

The scientific community has previously been driven by a reductionist standpoint that, while revealing, it may be limited to the point where a more holistic approach should be pursued in clarifying the glycolytic enzyme complex conundrum. The more recent adoption of a systems biology approach may represent a light at the end of the tunnel. Many studies have taken a reductionist approach in which the individual enzymes of interest were examined outside of the natural cellular matrix. The assumption that observation of a molecule’s behavior separated from its natural environment will be sufficient to understand its behavior within the cell is now known to be incomplete. It is clear that molecular behavior cannot be viewed so simply. The systems biology mindset is what is necessary for clarification of complicated dynamic interactions such as those involved in the putative glycolytic complex. Greater computational power available now could also improve *in silico* studies by permitting more parameters to be included and investigating more than two enzymes at a time. Building upon the studies done in muscle, using well-understood models for *in vivo* study such as *D. melanogaster*, and incorporation of holistic approaches may be what is needed to fill-in the gaps, corroborate previous studies and elucidate this elusive glycolytic enzyme complex.
